# Dialysis‐related practice patterns among hemodialysis patients with cancer

**DOI:** 10.1002/hsr2.46

**Published:** 2018-05-16

**Authors:** Hiroki Nishiwaki, Shingo Fukuma, Takeshi Hasegawa, Miho Kimachi, Tadao Akizawa, Shunichi Fukuhara

**Affiliations:** ^1^ Center for Innovative Research for Communities and Clinical Excellence Fukushima Medical University Fukushima Japan; ^2^ Division of Nephrology, Department of Internal Medicine Showa University Fujigaoka Hospital Yokohama Japan; ^3^ Human Health Sciences Kyoto University Graduate School of Medicine Kyoto Japan; ^4^ Institute for Health Outcomes and Process Evaluation Research (iHope International) Kyoto Japan; ^5^ Office for Promoting Medical Research Showa University Tokyo Japan; ^6^ Department of Healthcare Epidemiology, School of Public Health in the Graduate School of Medicine Kyoto University Kyoto Japan; ^7^ Division of Nephrology, Department of Medicine Showa University School of Medicine Tokyo Japan

**Keywords:** CKD‐MBD, erythropoiesis, hemodialysis, neoplasms, practice patterns, renal anemia

## Abstract

**Rationale, aims, and objectives:**

With the achievement of longevity in hemodialysis patients, the risk of comorbid cancer has begun to draw attention. In the present study, we examined dialysis‐related practice patterns and compared those patterns by cancer status.

**Methods:**

Using data from the Japan Dialysis Outcomes and Practice Patterns Study phase 4, we evaluated 2153 hemodialysis patients. Baseline cancer status for patients was separated into 3 categories: no cancer, cancer with recent treatment, and cancer without recent treatment. We then assessed variations among hemodialysis patients in dialysis‐related practice patterns, including anemia management, management of mineral and bone metabolism disorder, nutritional management, and dialysis treatment, by cancer status.

**Results:**

We observed both similarities and differences in dialysis‐related practice patterns among hemodialysis patients, by cancer status. Hemoglobin levels were largely similar for all cancer statuses, although erythropoiesis stimulating agents dose tended to be higher in hemodialysis patients with recent cancer treatment (multivariable adjusted mean difference of erythropoiesis stimulating agents dose: 5.4 × 10^3^ IU/L/month) than in those without cancer. Phosphorus and calcium levels were also similar. Nutrition statuses were similar among cancer statuses, as were dialysis therapies. These results suggested that physicians do not modulate their dialysis‐related practices based on whether or not a hemodialysis patient has cancer.

**Conclusion:**

Among long‐term facility‐based hemodialysis patients with cancer, we detected no statistically significant differences to suggest that cancer status affects hemodialysis practice regarding mineral and bone disorder management, nutritional management, and dialysis treatment. Facility‐based hemodialysis patients with recent cancer treatment, however, receive a higher dose of erythropoietin‐stimulating agent than those without cancer.

## 1 INTRODUCTION

The number of dialysis patients with cancer is on the rise, and discussion has started regarding optimum methods of dialysis treatment for these patients.^1^, ^2^ Because Japanese hemodialysis (HD) patients tend to be elderly, this patient population may be particularly prone to cancer, and the issue of appropriate treatment regimens will likely become relevant in Japan as in other countries.^3^, ^4^ However, optimum dialysis‐related practice patterns in these patients are unclear at present, and whether or not physicians alter treatment in HD patients according to cancer status merits investigation.

Dialysis‐related practice is multidisciplinary, including management for anemia, mineral and bone metabolism, and nutrition, and goes beyond simply the dialysis treatment itself. For example, while erythropoiesis‐stimulating agent (ESA) is widely used for anemia management in HD patients, the clinical guidelines for general patients with cancer recommend against the use of ESA in such situations, as ESA might adversely affect cancer progression.^5^, ^6^ Therefore, physicians have no clear basis upon which to develop an effective anemia management program in HD patients with cancer.

Once we determine if and how physicians presently alter their dialysis‐related practices for HD patients with cancer, we can begin to discuss how to improve the quality of care and clinical outcomes of this particular patient population. In the present study, to clarify the real‐world situation of these patients, we examined and compared the dialysis‐related practice pattern in patients with cancer with or without recent treatment, to those without cancer.

## 2 METHODS

### 2.1 Study design and data source

We conducted a cross‐sectional analysis using baseline data from the Japan Dialysis Outcomes and Practice Patterns Study (J‐DOPPS) phases 4 (2009‐2011). Details regarding the study design of J‐DOPPS were reported in a previous study.^7^ In the present study, we used laboratory data, medication data, comorbid condition data, and dialysis‐related practice data, which were all collected at baseline.

### 2.2 Ethical issues

J‐DOPPS complied with the Declaration of Helsinki, and all participants gave informed consent before enrollment. This study's conduct was approved by the Ethics Committee of Tokyo Women's Medical University (Approval No. 709, 1178, 1278, 1527, 1826, and 2143).

### 2.3 Study population

We included HD patients aged 18 or more who participated in J‐DOPPS. Patients without data on their cancer status were excluded.

### 2.4 Definition of cancer status

Information on the presence or absence of malignant tumors was collected at baseline in J‐DOPPS. Cancer status was divided into 3 categories: no cancer, cancer with recent treatment, and cancer without recent treatment. Presence or absence of cancer was measured at enrollment in J‐DOPPS. Recent treatment was defined as treatment for cancer within 1 year prior to the baseline evaluation. Cancer type was defined as solid tumor with metastasis, solid tumor without metastasis, myeloma, or leukemia.

### 2.5 Measurement of dialysis‐related practice

Dialysis‐related practice included anemia management, management of mineral and bone metabolism disorder (MBD), nutritional management, and dialysis treatment. These data were measured at baseline. For anemia management, we measured serum levels of hemoglobin (Hb) and ferritin; administration of ESA, including rHuEPO‐α (or β), darbepoetin‐α, and epoetin β pegol; ESA dose; erythropoietin resistance index (ERI); administration of intravenous iron; and blood transfusion. In accordance with the method of an earlier study, dose conversion from darbopoetin‐α or epoetin β pegol to rHuEPO‐α (or β) was performed using 1:200 ratios per week.^8^ ERI was evaluated as the weekly ESA dose per body weight (kg) per Hb. For management of MBD, we measured laboratory data (phosphorus, calcium, and intact parathyroid hormone), administration of phosphate binders (calcium carbonate, calcium acetate, sevelamer, and lanthanum carbonate), intravenous vitamin D receptor activators (calcitriol and maxacalcitol), and cinacalcet. We used the calcium values corrected for the serum albumin concentration.^9^ We defined hypercalcemia as serum calcium >10.5 mg/dL.^10^, ^11^ Nutritional management was assessed based on serum albumin levels and normalized protein catabolic rate (nPCR), which was calculated from predialysis and postdialysis blood urea nitrogen measurements. Further, practice variation of dialysis treatment was assessed based on single pool Kt/V, treatment time during each dialysis session, blood flow rate, and intradialytic weight loss.

### 2.6 Statistical analyses

We described patient characteristics by cancer status (no cancer, cancer with recent treatment, and cancer without recent treatment). Continuous data with a normal distribution were summarized as mean values (standard deviation), while continuous data with skewing were presented as medians (interquartile range) and categorical data as proportions. We also described dialysis‐related practice by cancer status. *P* values for differences in practices by cancer status were calculated using analysis of variance (ANOVA) or χ^2^ test. To account for the facility‐level clustering effect, we used a generalized estimating equation with robust variance.^12^, ^13^ We estimated the mean difference in the continuous data or odds ratios for dichotomous data in “cancer with and without recent treatment”, compared with the reference group of “no cancer.” We adjusted for age in the “age‐adjusted model” and for age, dialysis vintage, and comorbidities (coronary artery disease, congestive heart failure, cerebrovascular disease, diabetes, lung disease, neurologic disease, and peripheral vascular disease) in the “fully adjusted model”. To account for missing variables, we performed a complete‐case analysis. All analyses were conducted using STATA 14.1 (StataCorp, College Station, TX), with 2‐sided significance set at 0.05.

## 3 RESULTS

### 3.1 Patient characteristics by cancer status

Among 2153 HD patients, 84 (3.9%) had cancer without recent treatment and 68 (3.2%) had cancer with recent treatment (Table 1). In all groups, the mean age exceeded 60 years and nearly 40% in each group had diabetes. Patients who had cancer were more likely to be old and female than those without. In terms of cancer type, approximately 90% of cancers were solid tumors. Mean time since diagnosis was 7.8 years for those without recent treatment and 2.1 years for those with recent treatment; 56.6% of those without recent treatment and 13.6% of those with recent treatment had carry‐over cancer from predialysis chronic kidney disease.

**Table 1:** Patient characteristics by cancer status

	No Cancer		Cancer With Recent Treatment			
			No		Yes	
	n = 2001 (92.9%)		n = 84 (3.9%)		n = 68 (3.2%)	
Age, y (SD)	63.9	(12.5)	71.0	(9.1)	69.2	(10.7)
Sex, female, %	63.3		69.0		80.9	
Duration of dialysis, y (SD)	6.5	(7.4)	6.9	(6.8)	4.6	(6.8)
Cause of ESRD, n (%)						
Diabetic nephropathy	698	(34.9)	20	(23.8)	19	(27.9)
Glomerulonephritis	736	(36.8)	35	(41.7)	22	(32.4)
Nephrosclerosis	122	(6.1)	6	(7.1)	4	(5.9)
Others	445	(22.2)	23	(27.4)	23	(33.8)
Cancer type						
Solid tumor with metastasis, %	NA		15.5		21.0	
Solid tumor without metastasis, %	NA		79.8		67.7	
Myeloma, %	NA		1.2		9.7	
Leukemia, %	NA		3.6		1.6	
Time since diagnosis, y (SD)	NA		7.8	(6.6)	2.1	(2.7)
Time since latest treatment, y (SD)	NA		7.2	(6.4)	0.3	(0.5)
Carry‐over cancer to dialysis, %	NA		56.6		13.6	
Comorbidities						
Coronary heart disease, %	31.2		41.7		23.5	
Congestive heart failure, %	21.3		19.0		16.2	
Cerebrovascular disease, %	15.3		15.5		8.8	
Diabetes, %	39.7		36.1		37.3	
Lung disease, %	3.7		8.3		11.8	
Neurologic disease, %	9.6		6.0		2.9	
Peripheral vascular disease, %	18.3		23.8		16.2	

Abbreviations: ESRD, end‐stage renal disease; NA, not applicable.

### 3.2 Anemia management by cancer status

Table 2 shows the characteristics of anemia management by cancer status. The mean Hb level was similar between groups (*P* = .45, 1‐way ANOVA) and >10 g/dL in all groups. The mean ferritin level was different among the groups (*P* = 0.01, 1‐way ANOVA). The fully adjusted mean difference in ferritin level was 122.4 ng/mL in those with recent cancer treatment, compared with the reference group of “no cancer.” The mean ESA dose was higher in those with cancer than in the “no cancer” group. Among those with recent cancer treatment, the fully adjusted mean difference in ESA dose was 5.4 × 10^3^ IU/L/month compared with the “no cancer” group. The mean ESA resistance index was higher in patients with recent cancer treatment than in those without cancer (*P* < .04, fully adjusted generalized estimating equation). No significant differences were noted between groups in intravenous iron use or incidents of blood transfusion.

**Table 2:** Anemia management by cancer status^a^

	No Cancer		Cancer With Recent Treatment				*P* Value^b^
			No		Yes		
	n = 2001 (92.9%)		n = 84 (3.9%)		n = 68 (3.2%)		
Hemoglobin, g/dL (SD)	10.4	(1.3)	10.3	(1.2)	10.2	(1.3)	.45
Unadjusted mean difference, g/dL (95% CI)	Reference		−0.1	(−0.3 to 0.1) *P* = .39	−0.1	(−0.5 to 0.3) *P* = .58	
Age adjusted			0.0	(−0.2 to 0.2) *P* = .95	0.0	(−0.4 to 0.3) *P* = .87	
Fully adjusted			0.0	(−0.2 to 0.3) *P* = .79	0.0	(−0.4 to 0.3) *P* = .87	
Ferritin, ng/mL (IQR)	182.0	(97.0–340.0)	175.0	(101.2‐360.6)	200.2	(87.4‐405.0)	.01^c^
Unadjusted mean difference, ng/mL (95% CI)	Reference		35.6	(−12.0 to 83.3) *P* = .14	115.0	(−106.0 to 336.0) *P* = .31	
Age adjusted			28.6	(−20.8 to 77.9) *P* = .26	109.6	(−111.9 to 331.1) *P* = .33	
Fully adjusted			29.5	(−20.9 to 80.0) *P* = .25	122.4	(−108.6 to 352.3) *P* = .30	
TSAT, % (SD)	24.2	(11.0)	24.6	(9.4)	29.0	(15.1)	.16
Unadjusted mean difference, % (95% CI)	Reference		0.7	(−2.9 to 4.3) *P* = .71	5.1	(−1.9 to 12.1) *P* = .16	
Age adjusted			1.0	(−2.6 to 4.6) *P* = .58	5.2	(−1.8 to 12.1) *P* = .15	
Fully adjusted			0.7	(−3.0 to 4.5) *P* = .71	3.7	(−2.6 to 10.0) *P* = .25	
ESA use, % (SD)	88.5		86.9		94.1		.22
Unadjusted odds ratio (95% CI)	Reference		0.79	(0.44 to 1.42) *P* = .44	1.79	(0.83 to 3.88) *P* = .14	
Age adjusted			0.76	(0.43 to 1.37) *P* = .37	1.74	(0.81 to 3.75) *P* = .16	
Fully adjusted			0.8	(0.44 to 1.43) *P* = .44	2.3	(0.9 to 5.67) *P* = .08	
ESA dose, ×10^3^ IU/L/month (SD)	17.8	(16.8)	21.8	(18.9)	24.2	(17.5)	<.01^c^
Unadjusted mean difference, ×10^3^ IU/L/month (95% CI)	Reference		3.3	(0.3 to 6.3) *P* = .03	5.8	(2.0 to 9.7) *P* < .01	
Age adjusted			2.7	(−0.3 to 5.6) *P* = .08	5.4	(1.5 to 9.2) *P* < .01	
Fully adjusted			2.7	(−0.3 to 5.7) *P* = .08	5.4	(1.7 to 9.0) *P* < .01	
ERI (ESA resistance index) (SD)	8.6	(9.1)	11.1	(10.8)	11.1	(9.2)	<.01^c^
Unadjusted mean difference (95% CI)	Reference		2.0	(0.2 to 3.8) *P* = .03	2.3	(0.0 to 4.6) *P* = .05	
Age adjusted			1.5	(−0.3 to 3.3) *P* = .10	1.8	(−0.4 to 4.1) *P* = .12	
Fully adjusted			1.6	(−0.2 to 3.4) *P* = .08	2.3	(0.1 to 4.4) *P* = .04	
Intravenous iron use, %	22.0		22.6		26.5		.68
Unadjusted odds ratio (95% CI)	Reference		1.11	(0.67 to 1.83) *P* = .68	1.22	(0.67 to 1.78) *P* = .39	
Age adjusted			1.07	(0.65 to 1.78) *P* = .79	1.18	(0.76 to 1.83) *P* = .47	
Fully adjusted			1.05	(0.64 to 1.73) *P* = .84	1.13	(0.72 to 1.80) *P* = .59	
Blood transfusion, %	6.2		7.4		10.3		.42
Unadjusted odds ratio (95% CI)	Reference		1.21	(0.58 to 2.54) *P* = .61	1.73	(0.70 to 4.29) *P* = .24	
Age adjusted			0.95	(0.46 to 1.97) *P* = .90	1.44	(0.58 to 3.57) *P* = .44	
Fully adjusted			1.00	(0.48 to 2.1) *P* = .99	1.57	(0.60 to 4.12) *P* = .36	

Abbreviations: CI, confidence interval; ERI, erythropoietin resistance index = ESA dose/Body weight/Hemoglobin; ESA, erythropoiesis stimulating agent; IQR, interquartile range; SD, standard deviation; TSAT, transferrin saturation.

a Unadjusted and adjusted mean differences were shown as point estimate value with 95% CI accounting for the facility‐level clustering effect using generalized estimating equation, compared with the “no cancer” group.

b *P*‐value testing among all groups using Chi‐squared test for categorical variables or analysis of variance for continuous variables.

c *P* < .05.

### 3.3 MBD management by cancer status

Table 3 shows characteristics of MBD management by cancer status. The mean phosphorus level was similar between groups. The mean calcium level was also similar between groups, and few patients had hypercalcemia in any of the groups (no cancer: N = 83, 4.31%; cancer without recent treatment: N = 4, 5.00%; cancer with recent treatment: N = 1, 1.56%). No significant differences were noted between groups in intact parathyroid hormone levels, phosphate binder use, intravenous vitamin D receptor activator use, or cinacalcet use.

**Table 3:** Mineral and bone metabolism disorder management by cancer status^a^

	No Cancer		Cancer With Recent Treatment				*P* Value^b^
			No		Yes		
	n = 2001 (92.9%)		n = 84 (3.9%)		n = 68 (3.2%)		
Phosphorus, mg/dL (SD)	5.4	(1.4)	5.1	(1.4)	5.2	(1.5)	.09
Unadjusted mean difference, mg/dL (95%CI)	Reference		−0.3	(−0.6 to 0.1) *P* = .13	−0.1	(−0.6 to 0.3) *P* = .56	
Age adjusted			−0.1	(−0.5 to 0.2) *P* = .44	0.0	(−0.4 to 0.4) *P* = .91	
Fully adjusted			−0.2	(−0.5 to 0.2) *P* = .37	0.0	(−0.4 to 0.4) *P* = .89	
Calcium, mg/dL (SD)	8.9	(0.8)	8.7	(0.9)	8.5	(0.7)	.06
Unadjusted mean difference, mg/dL (95%CI)	Reference		−0.1	(−0.3 to 0.1) *P* = .31	−0.2	(−0.4 to 0.0) *P* = .03	
Age adjusted			−0.1	(−0.26 to 0.11) *P* = .40	−0.2	(−0.4 to −0.1) *P* = .04	
Fully adjusted			−0.1	(−0.2 to 0.1) *P* = .54	−0.1	(−0.3 to 0.1) *P* = .22	
Intact parathyroid hormone, pg/mL (IQR)	129.0	(66.0‐212.0)	169.5	(101.0‐214.5)	129.0	(65.0‐196.0)	.59
Unadjusted mean difference, pg/mL (95%CI)	Reference		16.0	(−17.8 to 49.8) *P* = .35	−18.1	(−46.7 to 10.5) *P* = .22	
Age adjusted			24.0	(−9.0 to 57.1) *P* = .15	−9.9	(−39,0 to 19.2) *P* = .51	
Fully adjusted			22.7	(−9.6 to 55.1) *P* = .17	−6.4	(−36.7 to 23.9) *P* = .68	
Phosphate binder use, %	71.2		75.0		67.6		.60
Unadjusted odds ratio (95% CI)	Reference		1.16	(0.74 to 1.81) *P* = .51	0.93	(0.52 to 1.67) *P* = .81	
Age adjusted			1.41	(0.90 to 2.22) *P* = .14	1.08	(0.60 to 1.95) *P* = .80	
Fully adjusted			1.35	(0.81 to 2.23) *P* = .25	1.03	(0.59 to 1.81) *P* = .91	
Intravenous vitamin D receptor activator use, %	24.2		20.2		26.5		.64
Unadjusted odds ratio (95% CI)	Reference		0.79	(0.16 to 1.62) *P* = .35	0.94	(0.55 to 1.60) *P* = .82	
Age adjusted			0.84	(0.51 to 1.39) *P* = .51	1.00	(0.58 to 1.71) *P* = 1.00	
Fully adjusted			0.79	(0.50 to 1.25) *P* = .31	0.99	(0.57 to 1.70) *P* = .96	
Cinacalcet use, %	7.9		4.8		2.9		.19
Unadjusted odds ratio (95% CI)	Reference		0.51	(0.16 to 1.62) *P* = .25	0.34	(0.08 to 1.49) *P* = .15	
Age adjusted			0.64	(0.20 to 2.06) *P* = .45	0.41	(0.09 to 1.78) *P* = .23	
Fully adjusted			0.55	(0.19 to 1.60) *P* = .27	0.41	(0.08 to 2.24) *P* = .31	

Abbreviations: CI, confidence interval; IQR, interquartile range; SD, standard deviation.

a Age‐adjusted and fully adjusted mean differences were estimated using a generalized estimating equation, compared with the “no cancer” group.

b *P*‐value testing among all groups using Chi‐squared test for categorical variables or analysis of variance for continuous variables.

### 3.4 Nutritional management by cancer status

Table 4 shows characteristics of nutritional management by cancer status. No significant difference in serum albumin or nPCR was noted in patients with cancer compared with those without cancer.

**Table 4:** Nutritional management by cancer status^a^

	No Cancer		Cancer With Recent Treatment				*P* Value^b^
			No		Yes		
	n = 2001 (92.9%)		n = 84 (3.9%)		n = 68 (3.2%)		
Serum albumin, g/dL (SD)	3.7	(0.5)	3.6	(0.4)	3.6	(0.5)	.05
Unadjusted mean difference, g/dL (95% CI)	Reference		−0.08	(−0.15 to −0.01) *P* = .03	−0.07	(−0.19 to 0.06) *P* = .31	
Age adjusted			−0.02	(−0.09 to 0.05) *P* = .58	−0.02	(−0.14 to 0.11) *P* = .79	
Fully adjusted			−0.02	(−0.09 to 0.05) *P* = .56	−0.03	(−0.15 to 0.09) *P* = .65	
Normalized protein catabolic rate, g/kg/d (SD)	0.95	(0.20)	0.93	(0.22)	0.96	(0.19)	.73
Unadjusted mean difference, g/kg/d (95% CI)	Reference		−0.01	(−0.06 to 0.04) *P* = .72	0.02	(−0.03 to 0.07) *P* = .43	
Age adjusted			0.00	(−0.05 to 0.05) *P* = .93	0.03	(−0.02 to 0.08) *P* = .26	
Fully adjusted			0.01	(−0.03 to 0.05) *P* = .68	0.04	(−0.01 to 0.10) *P* = .11	

Abbreviations: CI, confidence interval; SD, standard deviation.

a Age‐adjusted and fully adjusted mean differences were estimated using a generalized estimating equation, compared with the “no cancer” group.

b *P*‐value testing among all groups using analysis of variance for continuous variables.

### 3.5 Dialysis treatment by cancer status

Table 5 shows characteristics of dialysis treatment by cancer status. Dialysis treatment indicators of single pool Kt/V, treatment time during each dialysis session, blood flow rate, and intradialytic weight loss appeared to be similar across all groups.

**Table 5:** Dialysis treatment by cancer status^a^

	No Cancer		Cancer With Recent Treatment				*P* Value^b^
			No		Yes		
	n = 2001 (92.9%)		n = 84 (3.9%)		n = 68 (3.2%)		
Kt/V, single pool (SD)	1.34	(0.30)	1.38	(0.32)	1.34	(0.24)	.59
Unadjusted mean difference (95% CI)	Reference		0.00	(−0.56 to 0.51) *P* = .92	0.00	(−0.65 to 0.58) *P* = .91	
Age adjusted			0.00	(−0.06 to 0.05) *P* = .86	0.00	(−0.06 to 0.06) *P* = .88	
Fully adjusted			0.03	(−0.01 to 0.07) *P* = .18	0.05	(−0.01 to 0.10) *P* = .12	
Dialysis time, minutes (SD)	233.5	(30.7)	234.7	(20.4)	231.5	(27.2)	.82
Unadjusted mean difference (95% CI)	Reference		−1.4	(−5.1 to 2.3) *P* = .46	0.5	(−5.1 to 6.0) *P* = .88	
Age adjusted			0.7	(−3.3 to 4.7) *P* = .73	2.4	(−3.3 to 8.0) *P* = .42	
Fully adjusted			0.0	(−3.6 to 3.7) *P* = 1.00	3.7	(−1.8 to 9.3) *P* = .19	
Blood flow rate, ml/minute (SD)	202.3	(45.3)	198.5	(34.6)	203.2	(46.2)	.73
Unadjusted mean difference (95% CI)	Reference		−4.9	(−9.7 to 0.0) *P* = .05	1.5	(−4.8 to 7.7) *P* = .64	
Age adjusted			−1.4	(−6.2 to 3.3) *P* = .55	4.3	(−2.0 to 10.6) *P* = .18	
Full adjusted			−2.8	(−7.6 to 2.1) *P* = .26	0.7	(−5.3 to 6.8) *P* = .82	
Intradialytic weight loss, %	4.0		4.0		3.0		.49
Unadjusted mean difference (95% CI)	Reference		0.00	(0.00 to 0.01) *P* = .76	0.00	(−0.01 to 0.00) *P* = .05	
Age adjusted			0.00	(0.00 to 0.00) *P* = .58	0.00	(−0.01 to 0.00) *P* = .07	
Fully adjusted			0.00	(0.00 to 0.01) *P* = .49	0.00	(−0.01 to 0.00) *P* = .13	

Abbreviations: CI, confidence interval; SD, standard deviation.

a Age‐adjusted and fully adjusted mean differences were estimated using a generalized estimating equation, compared with the “no cancer” group.

b *P*‐value testing among all groups using analysis of variance for continuous variables.

## 4 DISCUSSION

We found both similar and different characteristics of dialysis‐related practice patterns among HD patients depending on their cancer status. Hb level was similar among all statuses, with ESA dose greater in HD patients with recent cancer treatment than in those without. Phosphorus levels and calcium levels were also similar across all statuses. Normalized protein catabolic rates were similar among all cancer statuses, as were dialysis therapies. These findings suggest that physicians did not modulate their dialysis‐related practices based on cancer status according to the patient's condition, among HD patients in Japan.

Controversy persists regarding ESA use for anemia management in patients with cancer. A previous systematic review revealed increased rates of death and thrombosis among ESA users with cancer.^14^ The clinical guideline and the US Food and Drug Administration, therefore, recommend against ESA use for anemia management in patients with cancer.^5^, ^6^ However, in the present study, we observed an increase of 5.4 × 10^3^ IU/L/month in ESA dose among HD patients who had recently received cancer treatment compared with those without cancer, after adjusting. The proportion of ESA use among patients with cancer exceeded 85% in the present study, which was higher than that noted in a previous study (37% in the CANDY study).^15^ Further, another descriptive study reported that the proportion of ESA use in HD patients with cancer was 90.0% based on Medicare data.^16^ These previous and present findings^15^, ^16^ suggest that there is no consensus in practice of ESA usage for HD patients with cancer.

Physicians might be administering a greater dose of ESA for HD patients with cancer in an effort to avoid unnecessary blood transfusions or iron replacement therapy.^17^, ^18^ However, although our results show that HD patients with recent cancer treatment tend to have higher ESA resistance than those without cancer, the rates of intravenous iron use and blood transfusion were similar between those with and without cancer. In Japan, no guidelines have been established for ESA use in cancer patients, and physicians are subject to restrictions on increasing the ESA dose, due to a bundling policy. We, therefore, hypothesize that physicians in the present study might have increased ESA doses in cancer patients in an effort to achieve the target Hb value, based on the guidelines for anemia management in dialysis patients.^19^, ^20^

In the present study, serum ferritin levels were different among cancer statuses. Some studies have reported that elevated serum ferritin was associated with malignancy.^21^, ^22^ In addition, an association has been reported between serum ferritin and treatment response or prognosis.^23^, ^24^, ^25^ To our knowledge, however, no evidence to suggest a relationship between recent treatment for cancer and serum ferritin has yet been found. Of note, the ERI in the patients with recent cancer treatment was higher than in those without cancer in our study. Patients with malignant neoplasms have a significantly higher ERI than those without.^26^ Some studies have reported that the transferrin saturation index is inversely related to ERI.^26^, ^27^ While the results of our study differ from those of previous studies, our findings were not adjusted through a multivariable analysis, and the trend for transferrin saturation was not statistically significant. Because the aim of our study was not to analyze the association between ERI and iron status, and because we lack a sufficient number of cases to perform a multivariable analysis of this association, we did not perform a multivariable regression analysis in our study.

In terms of MBD management, the proportions of phosphate binder use, intravenous vitamin D receptor activator use, and cinacalcet use did not markedly differ by cancer status. Phosphorus levels and calcium levels were also similar among cancer statuses. The mean values of phosphate, calcium, and intact‐parathyroid hormone were controlled to within the target ranges of the clinical guidelines, regardless of cancer status. Incidentally, hypercalcemia was found in approximately 1.5% to 5% of patients in each group.

Our results show no marked difference in the control of calcium levels between the patients with cancer and those without cancer. Because serum calcium levels tend to be higher because of hypercalcemia in patients with malignancy, calcium level control in patients with cancer is generally difficult.^10^ However, the calcium levels were well controlled in the patients with cancer in the present study, suggesting that clinicians should be able to control the calcium level regardless of the presence of cancer.^28^

Neither nutritional management nor dialysis treatment differed markedly among cancer statuses. Although both HD and cancer patients have been reported to be at risk for having low nutritional status,^29^, ^30^ we observed no substantial effect of cancer on any nutritional indicators. Appetite loss and nausea are known to be adverse effects in cancer patients undergoing chemotherapy or radiation therapy, but we did not observe any marked difference in nutritional indicators between cancer status groups.^31^ The nutritional therapies for HD patients with cancer will need to be examined in a future study, though, as we did not gather such data in the present study.

In terms of dialysis treatment, Japanese HD patients receive moderate dialysis therapy at a low blood flow rate, with a long dialysis time and low intradialytic weight in comparison with other countries.^32^ Patients with cancer received similar dialysis therapy, perhaps because such patients are generally tolerant of these moderate dialysis therapies. However, the number of patients who withdrew from maintenance HD was difficult to determine using our data, although a previous study reported that some patients did withdraw from HD therapy after being diagnosed with cancer.^33^

Among baseline characteristics, a number of differences were seen between patients with and without cancer; among these, patients with cancer tended to be older and more commonly female than those without cancer. In general, older patients tend to have a higher risk of cancer. Similar trends were found in end‐stage renal disease (ESRD) patients: one study reported a higher standardized incident rate of cancer in ESRD patients aged over 40 years than in those under 40 years of age,^34^ while a study in Taiwan reported that the risk of cancer in female patients with HD was higher than that in males.^35^ These differences in baseline characteristics may explain our present findings.

Several strengths to the present study warrant mention. First, 2‐stage stratified random sampling was used in the J‐DOPPS cohort. Therefore, this study involves a representative cohort of Japanese HD patients. Second, we had access to detailed data from this cohort on multidisciplinary dialysis‐related practice patterns, including anemia management, MBD management, nutritional management, and dialysis treatment. Third, to our knowledge, this is the first study to examine variations in dialysis‐related practice patterns based on cancer status.

However, several limitations should also be mentioned. First, we only included patients who received long‐term HD therapy at a facility. Therefore, our results may not be applicable to other ESRD populations, such as predialysis patients, peritoneal dialysis patients, and home dialysis patients. In addition, we only included Japanese HD patients. Second, other factors might have affected the dialysis‐related treatment. For example, mean age differed among the cancer status groups (Table 1), and dialysis‐related therapeutic regimens might differ by age. To account for the difference in age between groups, we assessed the age‐ and fully adjusted differences in practice between groups. We, therefore, believe that our adjusted findings are still of interest. Third, these data also included no information on the cancer activity, such as whether the cancer was active or in remission, phenotype of cancer, or cancer stages, factors that are extremely important for predicting the prognosis and determining appropriate dialysis practices. Because most of the J‐DOPPS facilities were HD clinics, follow‐up of patients admitted to a hospital with cancer was difficult. In addition, the cancer without recent treatment group might have included both patients whose cancer had completely healed a long time ago and those in whom cancer had been diagnosed but not yet treated. Fourth, we were unable to assess the effect of the practice pattern, including therapy for anemia and chronic kidney disease–mineral and bone disorder, on the cancer status. Fifth, our population included a relatively small number of patients with cancer (n = 152). This small sample size prevented examination of the association between practice patterns and clinical outcome in patients with cancer. Further studies with larger populations of cancer patients will be needed in the future. Sixth, an additional standardized screening for cancer for our study was not conducted, so the evaluation of the presence of cancer depended on physicians and facilities. These limitations might have resulted in some degree of bias in the present study. Finally, we use serum albumin and nPCR as indicators of the nutritional status. Several other nutritional indicators have been proposed, such as the geriatric nutritional risk index, subjective global assessment, and phase angle based on a bioelectrical impedance analysis, but we were unable to obtain these indexes in our cohort.

Among patients with cancer who receive facility‐based HD treatment in Japan, we detected no statistically significant differences to suggest that cancer status affected HD practice regarding MBD management, nutritional management, and dialysis treatment. Facility‐based HD patients with cancer receive a higher ESA dose than those without cancer.

## FUNDING

This study was supported by Kyowa Hakko Kirin. The funder had no role in study design, data collection and analysis, decision to publish, or preparation of the manuscript.

## CONFLICT OF INTEREST

Nishiwaki H. received grants from Kyowa Hakko Kirin. Fukuma S. has acted as a scientific advisor to Kyowa Hakko Kirin. Fukuhara S. has acted as a scientific advisor to and received grants from Kyowa Hakko Kirin. Akizawa T. has consultancy agreements with Astellas Pharma, AbbVie, Kyowa Hakko Kirin, Bayer HealthCare Pharmaceuticals, GlaxoSmithKline, JT Pharmaceuticals Corporate, Kissei Pharmaceutical, Nipro Medical Corporation, Ono Pharmaceutical, and is a member of the Astellas' advisory panel. Akizawa T. is also a scientific advisor of Bayer HealthCare Pharmaceuticals and a member of the speaker's bureau of Bayer HealthCare Pharmaceuticals, Kyowa Hakko Kirin, Chugai Pharmaceutical, Torii Pharmaceutical, Fuso Pharmaceutical Industries, and Teijin Pharma. Hasegawa T has consultancy agreements with Kyowa Hakko Kirin. These conflicts did not affect the design, results, or conclusions of the study.

## AUTHOR CONTRIBUTIONS

Conceptualization: Hiroki Nishiwaki, Takeshi Hasegawa

Formal analysis: Shingo Fukuma, Miho Kimachi

Methodology: Hiroki Nishiwaki, Takeshi Hasegawa

Supervision: Tadao Akizawa, Shunichi Fukuhara

Writing—original draft preparation: Hiroki Nishiwaki, Shingo Fukuma, Miho Kimachi

Writing—review and editing: Takeshi Hasegawa, Tadao Akizawa
